# Editorial: Health and production issues in smallholder pig farming

**DOI:** 10.3389/fvets.2023.1320982

**Published:** 2023-12-18

**Authors:** Anne Conan, Elizabeth A. J. Cook, Maria José Hötzel, Beatriz Martínez-López

**Affiliations:** ^1^Centre for Applied One Health Research and Policy Advice, City University of Hong Kong, Kowloon, Hong Kong SAR, China; ^2^Animal and Human Health Program, International Livestock Research Institute, Nairobi, Kenya; ^3^Laboratório de Etologia Aplicada e Bem-Estar Animal, Universidade Federal de Santa Catarina, Florianópolis, Brazil; ^4^Center for Animal Disease Modelling and Surveillance (CADMS), School of Veterinary Medicine, University of California, Davis, Davis, CA, United States

**Keywords:** swine, extensive farming, value chains, infectious diseases, zoonoses, antimicrobial usage, gender analysis

Pig production ranks as the second largest livestock industry by animal numbers worldwide, with an ever-increasing demand for pork among consumers (1, 2). Concurrently, farming systems have evolved into intensive or semi-intensive raising practices over the past few decades. Research efforts in swine production have extensively studied indicators of pig health and production in these systems, along with the impact of diseases, surveillance systems and interventions to address production issues on these farms. On the other hand, smallholder pig farming (Figure 1) continues to exist in parallel due to economic and cultural reasons, even increasing as pasture pig operations have become the choice for animal welfare advocates. However, pig health and production indicators are less well-documented in these extensive systems because of their limited impact on global pork trade.

**Figure 1:**
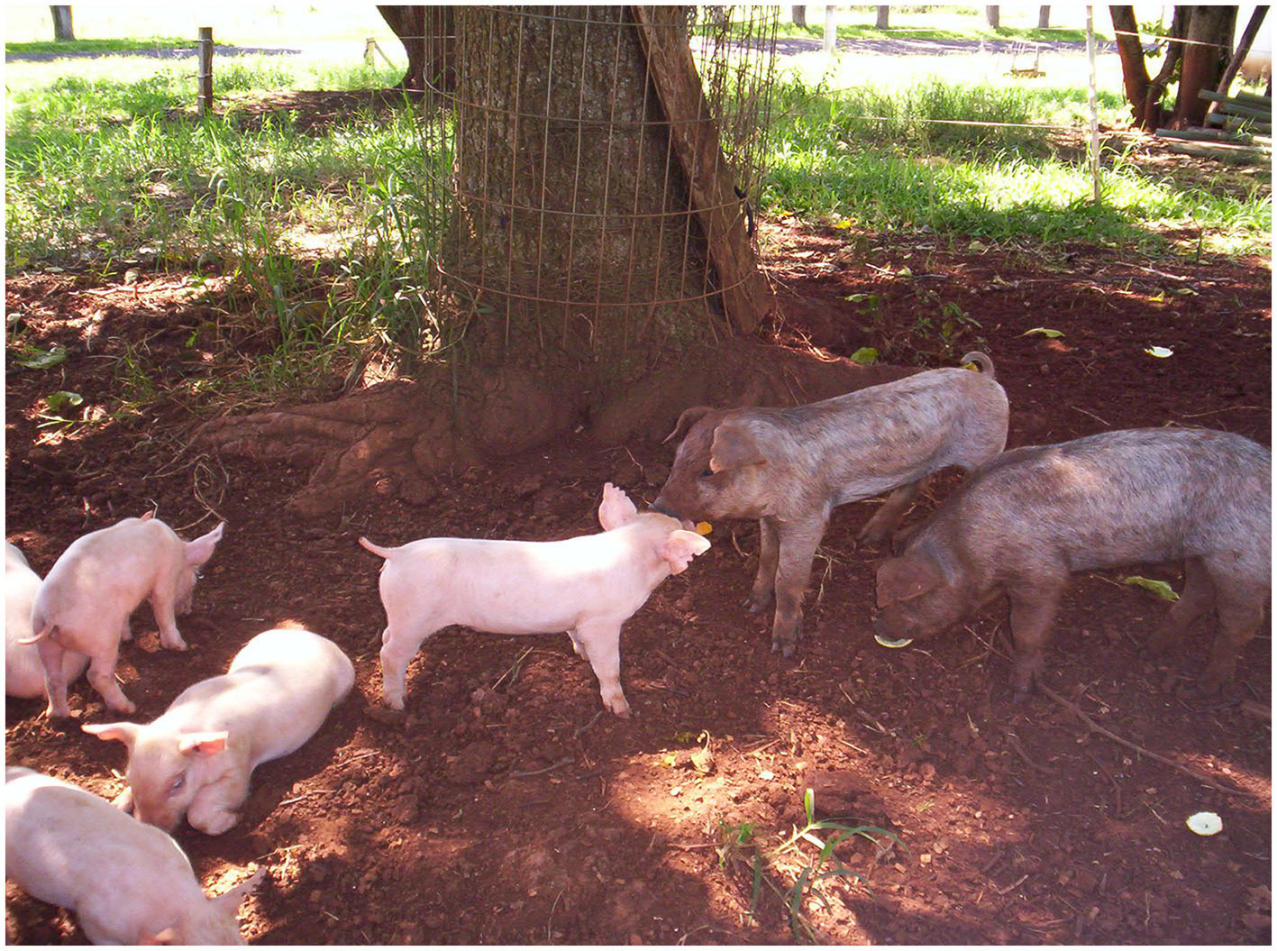
Outdoor raised pigs (Exotic Landrace and local Moura breed) in a smallholder demonstration farm (Brazil). Credit: MH.

With the re-emergence and global spread of African swine fever (ASF) virus beyond Africa, smallholder farms have been pointed out as key players to the spread and mantainance of this swine pandemic. While the exact role of smallholder farms in ASF virus epidemiology is debatable, these allegations placed considerable pressure on smallholder farmers to modify their practices. Nevertheless, it's important to acknowledge that ASF is not the sole challenge faced by smallholders. It is imperative to understand the broader challenges and opportunities that smallholder farmers encounter in order to ultimately improve pig health and prevent infectious diseases. Regrettably, research studies to understand these systems are scarce, and recommendations for enhancing health and production lack an evidence-based foundation and are usually unfeasible given the socio-economic reality of these operations.

The primary objective of this Research Topic was to compile studies that describe the diversity of smallholder pig farming and the production and health issues experienced by pig smallholders. The articles gathered in this Research Topic cover various geographical regions (East Africa, South Asia, and South-East Asia). Still, they all highlight common themes and challenges: market access and the impact of infectious diseases on trade, biosecurity and feed access, access to veterinary services, awareness of occupational and food-borne pathogens, and antimicrobial usage.

Despite local market access, the pork value chain drives the demand for pork and the management of smallholder pig farms. Nguyen-Thi-Duong et al. conducted a value-chain mapping with a gender-focus analysis using semi-structured interviews and focus groups involving input suppliers, pig producers, slaughterhouse owners, pork processors, pork retailers, and pork consumers. Their findings revealed low concern regarding occupational health and a misperception of food safety among both genders. Conversely, Ngwili et al. demonstrated that the presence or absence of cysticercosis cysts (a food safety hazard) significantly influenced trade intensity, resulting in the rejection of pigs by traders at purchase, condemnation of pig carcasses, and rejection of raw pork by consumers. These authors studied the value chains in four different districts of Uganda, using key informant interviews and focus group discussions, focusing on the impact of cysticercosis cysts on trade. They also highlighted the lack of control measures against infectious diseases throughout the entire value chain. Furthermore, they noted deep-rooted gender inequality, although, Nguyen-Thi-Duong et al.'s study did not find a gender inequality perception among participants despite gender-based variations in stakeholder duties and farming knowledge (with better knowledge observed among male farmers). Millar et al. examined a specific segment of the value chain but chose a different study population (farmers, communities, and veterinary services). Their qualitative study included focus groups with farmers, community engagement training study, and an evaluation of the community engagement program. They also considered gender inequality in their study methodology and found results contrary to Nguyen-Thi-Duong et al., with female participants having better knowledge of pig diseases.

To prevent and control the introduction of pathogens into farms, the implementation of biosecurity measures is necessary. Singh et al. conducted a cross-sectional study in India to evaluate biosecurity practices among smallholder pig farmers. They interviewed 1,000 pig producers in urban and rural areas (where no ASFV occurred) and described that the majority of respondents reported frequent morbidity with clinical signs such as fever, diarrhea, inappetence, and death in their pigs during the past year. Basic biosecurity measures such as fencing, footbaths, or quarantine of new animals were not widely adopted. About biosecurity, Chege et al. explored the impact of latrine construction on cysticercosis occurrence. By comparing the results of a recent cross-sectional study with another study conducted before latrine construction, they observed a decrease in lingual cyst prevalence. They also found that allowing pigs to roam freely increased the risk of cysticercosis on farms. Unfortunately, implementing strict biosecurity measures may lead to other challenges for farmers, such as leg wounds in tethered pigs. Millar et al., Singh et al., and Chege et al. all reported the use of swill or kitchen scraps, which is more economically viable than recommended commercial pig feed.

Despite farmers reporting a range of infectious diseases, including zoonoses and food-borne pathogens, access to veterinary services remains limited, failing to adequately support farmers in addressing these issues. Lack of trust in veterinary services, distance from veterinary providers, and limited knowledge of diseases and treatments are recurring topics in the papers. This can partly explain the indiscriminate use of antimicrobials. Ting et al. conducted a cross-sectional study involving face-to-face interviews to assess knowledge and practices related to antibiotic use and antibiotic resistance. They reported a low level of antibiotic knowledge but found no instances of antibiotics being used for disease prevention or growth promotion.

The collection of articles in this Research Topic highlights the diverse challenges faced by smallholder pig farmers. We encourage further research in areas such as the pork value chain, farm biosecurity, access to veterinary services by these smallholder pig farms, and expansion of local professional expertise (3). It's important to note that most of these studies relied on interview methods, with only a few collecting samples for diagnostic confirmation of disease. Investigating the occurrence of infectious diseases in conjunction with the implementation of questionaires in these systems is crucial. Furthermore, given the presence of zoonoses, food borne pathogens and antimicrobial resistance in these smallholder systems and the frequent interactions with other livestock and wildlife, future research on pig farming in smallholder settings should adopt an integrated One Health approach. Ultimately, this approach will benefit the livelihoods of pig farmers, enhance the health and well-being of pigs, improve the overall pork trade and safeguard public health.

## Author contributions

AC: Conceptualization, Writing—original draft. EC: Writing—review & editing. MH: Writing—review & editing. BM-L: Writing—review & editing.
